# *In vitro* susceptibility and provisional epidemiological cutoff values for eight antifungal agents against *Trichophyton mentagrophytes* and *Trichophyton interdigitale*: a multicenter study from China

**DOI:** 10.1128/jcm.00567-26

**Published:** 2026-08-21

**Authors:** Hailin Zheng, Wenting Xie, Yinggai Song, Cunwei Cao, Meng Fu, Biling Dong, Xiaodong Wang, Xue Kong, Naicen Ge, Huan Mei, Ruoyu Li, Kaisu Pan, Yanyang Guo, Zhongsheng Tong, Paride Abliz, Xiaodong She, Weida Liu, Guanzhao Liang, Xiaofang Li

**Affiliations:** 1 Department of Medical Mycology, Hospital for Skin Diseases, Institute of Dermatology, Chinese Academy of Medical Sciences & Peking Union Medical College 70575, Nanjing, China; 2 Jiangsu Key Laboratory of Molecular Biology for Skin Diseases and STIs, Nanjing, China; 3 CAMS Collection Centre of Pathogen Microorganisms-D (CAMS-CCPM-D), Nanjing, China; 4 Department of Dermatology, Peking University First Hospital 26447, Beijing, China; 5 Department of Dermatology, the First Affiliated Hospital of Guangxi Medical University 117742, Nanning, Guangxi, China; 6 Department of Dermatology, Xijing Hospital, Fourth Military Medical University 12644, Xi'an, Shaanxi, China; 7 Department of Dermatology, Wuhan No.1 Hospital 593237 , Wuhan, Hubei, China; 8 Department of Dermatology, First Affiliated Hospital of Xinjiang Medical University 159427, Urumqi, Xinjiang, China; University of Utah Health Medicine, Salt Lake City, Utah, USA

**Keywords:** *Trichophyton mentagrophytes*, *Trichophyton interdigitale*, epidemiological cutoff values, antifungal susceptibility, CLSI M38

## Abstract

Dermatophytosis is a highly prevalent superficial fungal infection, yet antifungal susceptibility testing results remain difficult to interpret because clinical breakpoints are unavailable, and epidemiological cutoff values (ECVs) are still limited for most dermatophyte–drug combinations. We conducted a multicenter study of 305 *Trichophyton mentagrophytes* and *Trichophyton interdigitale* isolates collected between 2019 and 2024 at six tertiary hospitals across geographically diverse regions of China. Antifungal susceptibility testing was performed using the CLSI M38 broth microdilution method under standardized quality control, and provisional ECVs were derived following CLSI M57 guidance. For *T. mentagrophytes*, fluconazole showed a non-unimodal MIC distribution and was not assigned a provisional ECV. The remaining seven agents yielded ECVs of 1 µg/mL for ciclopirox olamine and griseofulvin, 0.5 µg/mL for itraconazole, 0.06 µg/mL for voriconazole, 0.25 µg/mL for posaconazole and amorolfine, and 0.016 µg/mL for terbinafine. For *T. interdigitale*, fluconazole showed a complex non-unimodal distribution, whereas itraconazole and posaconazole showed unstable low-MIC shoulders precluding ECV estimation. The remaining five agents yielded ECVs of 1 µg/mL for ciclopirox olamine, 0.25 µg/mL for griseofulvin, 0.12 µg/mL for voriconazole and amorolfine, and 0.016 µg/mL for terbinafine. Isolates above the provisional ECVs were uncommon for most combinations (≤2.5%), except for griseofulvin against *T. interdigitale* (20.5%). Overall, the two species showed distinct MIC distribution profiles, underscoring the value of species-level analysis and continued surveillance. These multicenter CLSI-based data and provisional ECVs provide a contemporary baseline for species-specific interpretation of dermatophyte susceptibility data in China.

**IMPORTANCE:**

Reliable interpretation of dermatophyte antifungal susceptibility testing remains limited because clinical breakpoints and epidemiological cutoff values (ECVs) are unavailable for most dermatophyte–drug combinations. This is particularly important for the *Trichophyton mentagrophytes*/*Trichophyton interdigitale* species complex, which is increasingly implicated in refractory dermatophytosis and may display species-specific susceptibility patterns. In this multicenter study, we established provisional epidemiological cutoff values for most tested species–agent combinations and showed that these two closely related species differ in MIC distribution profiles and in the detection of non-wild-type isolates. These data provide practical laboratory thresholds for recognizing isolates with reduced susceptibility, highlight the limitations of complex-level interpretation, establish a contemporary baseline for dermatophyte susceptibility surveillance in China, and may help support laboratory-informed antifungal decision-making.

## INTRODUCTION

Dermatophytosis is the most common superficial fungal infection worldwide and has become increasingly important in clinical practice because of its high prevalence, broad anatomic involvement, and impact on quality of life (1, 2). Although traditionally regarded as a readily treatable superficial infection, it has gradually been associated with refractory, recurrent, and persistent disease (3, 4). Of particular concern are infections caused by the *Trichophyton mentagrophytes*/*Trichophyton interdigitale* species complex (TMTISC), which has drawn growing attention. In recent years, members of this complex, particularly *Trichophyton indotineae*, have been increasingly recognized as major causes of difficult-to-treat dermatophytosis, often presenting as extensive disease involving large areas of the body, especially in South Asia, where their emergence has been associated with widespread terbinafine resistance and a shift in the dominant dermatophyte species in India (5–8). As the epidemiology of dermatophyte infections and their antifungal susceptibility profiles continues to evolve, accurate species identification and standardized susceptibility testing are becoming increasingly relevant to patient care (9).

Current treatment options for dermatophytosis include allylamines, azoles, hydroxypyridones, morpholines, and griseofulvin, but antifungal activity is not uniform across dermatophyte species (10). Recent studies from China and elsewhere have documented elevated MICs or reduced susceptibility to selected agents among members of TMTISC (11, 12). *T. mentagrophytes* and *T. interdigitale* are clinically important species that may differ in anatomic distribution, patient characteristics, and antifungal susceptibility profiles (13). In addition, both species usually produce abundant microconidia *in vitro*, facilitating preparation of standardized inocula for broth microdilution testing according to CLSI M38 (14). These characteristics make them more suitable for species-level analysis of MIC distributions and for the development of standardized laboratory thresholds.

Interpretation of dermatophyte MIC data remains limited because clinically validated breakpoints are unavailable for all dermatophyte–antifungal combinations. In this setting, epidemiological cutoff values (ECVs/ECOFFs) provide a practical means of distinguishing wild-type from non-wild-type isolates and thus support *in vitro* resistance monitoring (15). At present, no official ECVs or ECOFFs are available for dermatophyte, except for tentative European Committee on Antimicrobial Susceptibility Testing (EUCAST) ECOFFs proposed for selected combinations involving *Trichophyton rubrum* and *T. indotineae* (16). CLSI M38 methodology has long been used for dermatophyte antifungal susceptibility testing, whereas EUCAST introduced a modified dermatophyte methodology and tentative ECOFFs in 2021 (14, 17). Although both systems provide approaches, important methodological differences exist between CLSI and EUCAST, including culture medium composition, inoculum concentration, incubation temperature, and duration, which complicate direct comparison of CLSI-derived ECVs and EUCAST-derived ECOFFs (14, 17). CLSI M38 remains the most widely recognized broth microdilution reference method for filamentous fungi, and CLSI M57 provides the principles for establishing ECVs from CLSI-generated MIC data (14, 18). However, multicenter CLSI M38-based ECV data for well-identified *T. mentagrophytes* and *T. interdigitale* isolates remain lacking (19).

In this study, we analyzed clinical isolates of *T. mentagrophytes* and *T. interdigitale* collected between 2019 and 2024 at six tertiary hospitals in China. Under a unified quality control strategy, each participating center performed broth microdilution testing according to the CLSI M38 method against eight commonly used antifungal agents: terbinafine, fluconazole, itraconazole, voriconazole, posaconazole, griseofulvin, ciclopirox olamine, and amorolfine. We then characterized species-specific MIC distributions and established provisional epidemiological cutoff values according to CLSI M57 principles. We aimed to generate multicenter baseline data from China for species-stratified dermatophyte resistance monitoring and to provide laboratory thresholds that may help identify potential non-wild-type isolates and inform antifungal treatment decisions.

## RESULTS

### Clinical types of infections

**Fig 1:**
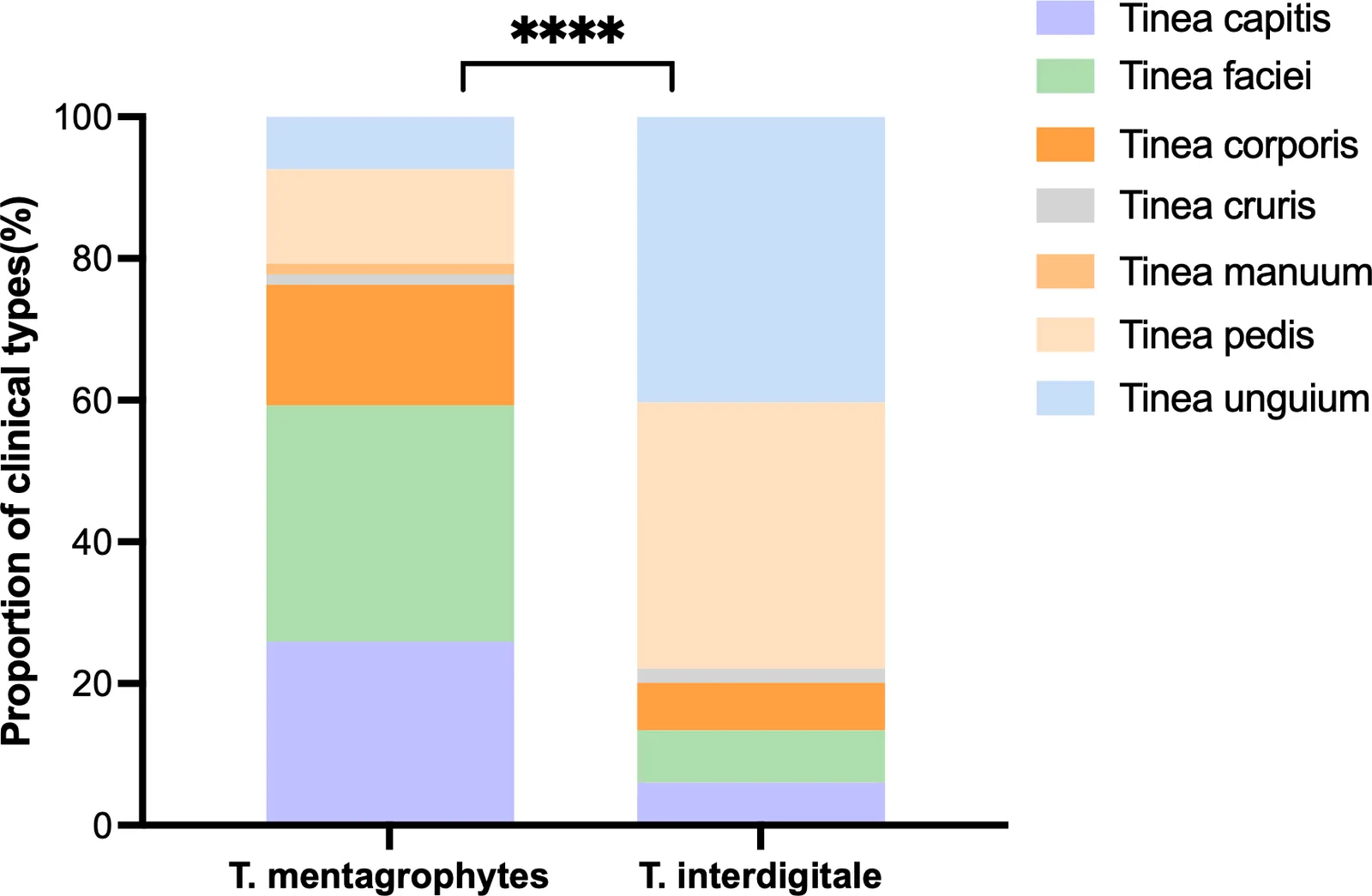
Proportion of clinical types among patients infected with *T. mentagrophytes* (*n* = 135) and *T. interdigitale* (*n* = 149). ****, *P* < 0.0001.

A total of 305 isolates were included in the ECV analysis, comprising 153 *T. mentagrophytes* and 152 *T. interdigitale* isolates collected from six hospitals in China. Species-level classification was supported by ITS-based phylogenetic comparison with published TMTISC reference sequences (Fig. S1). Because clinical data were unavailable for some isolates, analyses of clinical presentation were based on available cases. The distribution of clinical infection types differed significantly between the two species (*χ*^2^ test, *P* < 0.0001; Fig. 1). *T. mentagrophytes* was most recovered from tinea faciei (45/135, 33.33%) and tinea capitis (35/135, 25.93%), followed by tinea corporis (23/135, 17.04%), tinea pedis (18/135, 13.33%), tinea unguium (10/135, 7.41%), tinea manuum (2/135, 1.48%), and tinea cruris (2/135, 1.48%). Differently, *T. interdigitale* was isolated predominantly from tinea unguium (60/149, 40.27%) and tinea pedis (56/149, 37.58%), with fewer isolates from tinea faciei (11/149, 7.38%), tinea corporis (10/149, 6.71%), and tinea capitis (9/149, 6.04%). These analyses solely provide descriptive epidemiological context and were not used for ECV determination.

### Provisional epidemiological cutoff values for *T. mentagrophytes*

**Fig 2:**
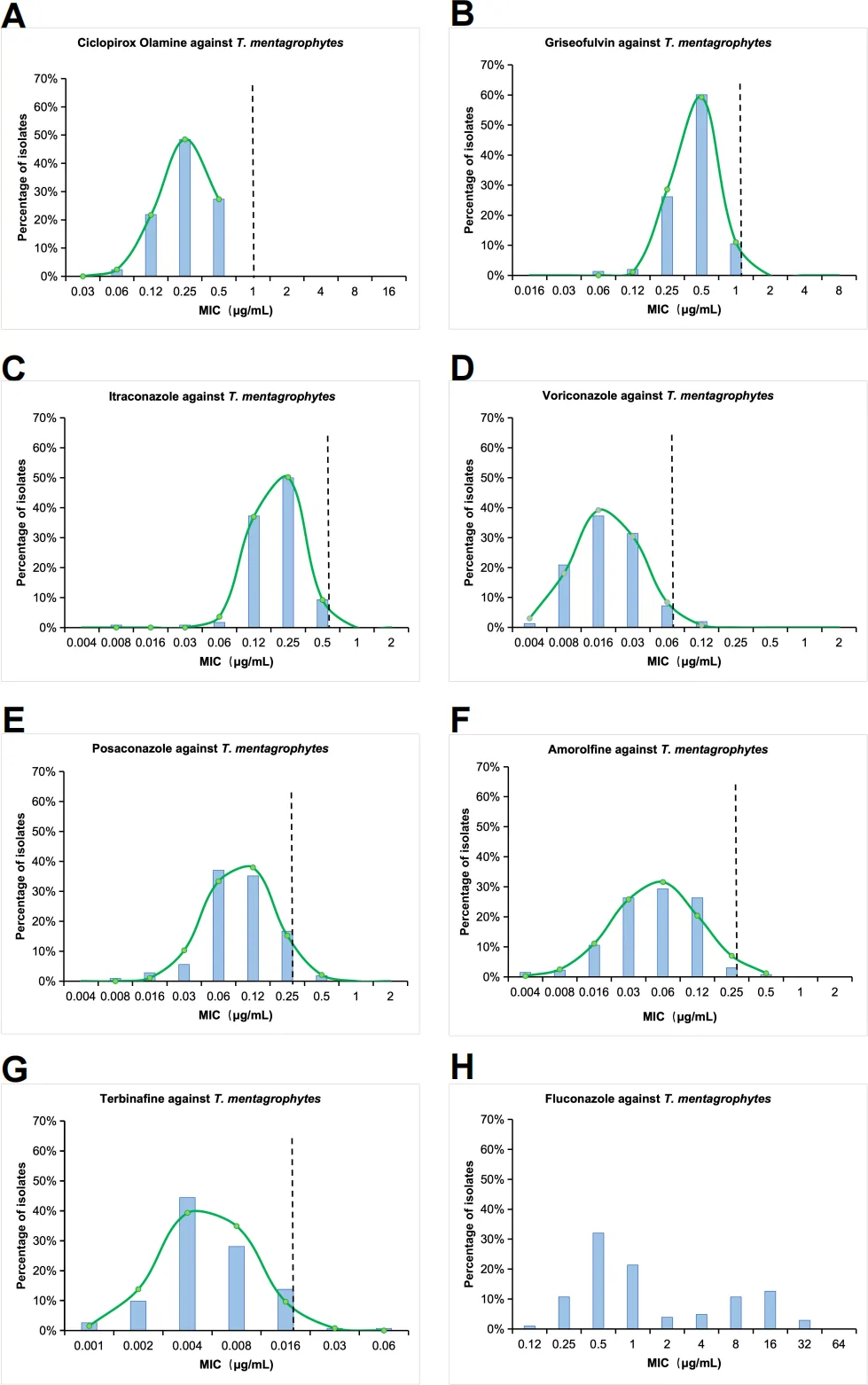
(**A–G**) MIC distributions and provisional epidemiological cutoff values (ECVs) for seven antifungal agents against *T. mentagrophytes*. (**H**) MIC distribution of fluconazole; no provisional ECV was assigned.

*In vitro* susceptibility data (Table S1) for eight antifungal agents against *T. mentagrophytes* were generated using the CLSI M38 broth microdilution method (14). Table 1 summarizes the MIC ranges and key MIC parameters, and representative MIC distributions are shown in Fig. 2A through H. Seven of the eight MIC distributions were compatible with unimodal wild-type modeling and were therefore considered suitable for provisional ECV calculation. In contrast, fluconazole showed a non-unimodal MIC distribution with a distinct high-MIC subpopulation and was not retained for provisional ECV estimation. Using ECOFFinder (version 2.1) according to CLSI M57 principles, provisional ECVs at the 97.5% cutoff were estimated at 1 µg/mL for ciclopirox olamine and griseofulvin; 0.5 µg/mL for itraconazole; 0.06 µg/mL for voriconazole; 0.25 µg/mL for posaconazole and amorolfine; and 0.016 µg/mL for terbinafine. Isolates above the provisional ECV were identified for voriconazole (1.9%), posaconazole (1.8%), amorolfine (0.7%), and terbinafine (1.3%), whereas all isolates remained at or below the provisional ECVs for ciclopirox olamine, griseofulvin, and itraconazole.

**TABLE 1:** MIC parameters and provisional epidemiological cutoff values of eight antifungal agents against *T. mentagrophytes* and *T. interdigitale^a^*

Antifungal drug	Species	No. of participating centers (isolates tested)	MIC range (μg/mL)	Modal MIC (μg/mL)	Geometric mean MIC (μg/mL)	MIC50 (μg/mL)	MIC90 (μg/mL)	ECV (μg/mL)	NWT isolates
Fluconazole	*T. mentagrophytes*	4 (103)	0.12–32	0.5	1.46	1	16	NA	NA
	*T. interdigitale*	6 (152)	0.12–32	8	3.33	8	16	NA	NA
Ciclopirox olamine	*T. mentagrophytes*	5 (128)	0.06–0.5	0.25	0.25	0.25	0.5	1	0.00%
	*T. interdigitale*	5 (127)	0.03–0.5	0.25	0.17	0.25	0.25	1	0.00%
Griseofulvin	*T. mentagrophytes*	6 (153)	0.06–1	0.5	0.42	0.5	1	1	0.00%
	*T. interdigitale*	5 (122)	0.016–1	0.12	0.14	0.12	0.5	0.25	20.50%
Itraconazole	*T. mentagrophytes*	4 (118)	0.008–0.5	0.25	0.19	0.25	0.25	0.5	0.00%
	*T. interdigitale*	4 (120)	0.004–0.5	0.25	0.14	0.25	0.25	NA	NA
Voriconazole	*T. mentagrophytes*	6 (153)	0.004–0.12	0.016	0.02	0.016	0.03	0.06	1.90%
	*T. interdigitale*	5 (135)	0.004–0.12	0.03	0.03	0.03	0.06	0.12	0.00%
Posaconazole	*T. mentagrophytes*	4 (108)	0.008–0.5	0.06	0.1	0.12	0.25	0.25	1.80%
	*T. interdigitale*	4 (120)	0.004–0.5	0.12	0.09	0.12	0.25	NA	NA
Amorolfine	*T. mentagrophytes*	5 (133)	0.004–0.5	0.06	0.05	0.06	0.12	0.25	0.70%
	*T. interdigitale*	4 (112)	0.004–0.5	0.03	0.04	0.03	0.12	0.12	1.80%
Terbinafine	*T. mentagrophytes*	6 (153)	0.001–0.06	0.004	0.005	0.004	0.016	0.016	1.30%
	*T. interdigitale*	6 (152)	0.001–0.03	0.004	0.003	0.004	0.008	0.016	1.30%

^*a*^ MIC, minimum inhibitory concentration; ECV, epidemiological cutoff value; NA, not assigned because the MIC distribution was non-unimodal and unsuitable for provisional ECV estimation; NWT, non-wild-type.

### Provisional epidemiological cutoff values for *T. interdigitale*

**Fig 3:**
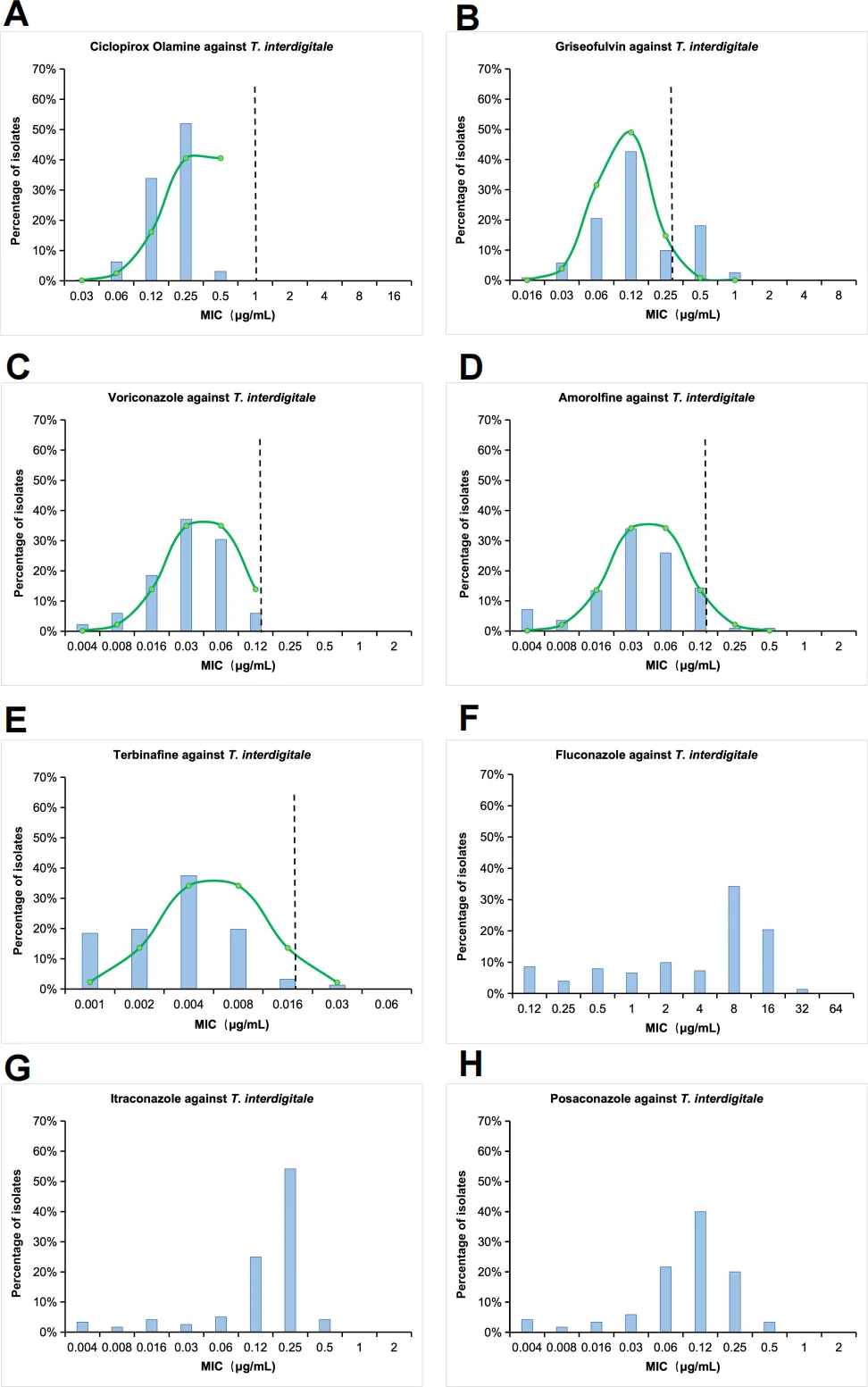
(**A–E**) MIC distributions and provisional epidemiological cutoff values (ECVs) for five antifungal agents against *T. interdigitale*. (**F–H**) MIC distributions of fluconazole, itraconazole, and posaconazole for *T. interdigitale*; no provisional ECVs were assigned.

Table 1 also outlines the MIC ranges and key MIC parameters for *T. interdigitale*. Unlike *T. mentagrophytes*, *T. interdigitale* exhibited greater heterogeneity in MIC distributions across agents. Fluconazole showed a complex, non-unimodal MIC distribution and was therefore not retained for provisional ECV derivation. Itraconazole and posaconazole showed low-MIC shoulders centered at lower MICs and were therefore not considered sufficiently stable for robust species-level ECV determination. The remaining five combinations were retained for ECOFFinder analysis. Provisional ECVs at the 97.5% cutoff were estimated at 1 µg/mL for ciclopirox olamine; 0.25 µg/mL for griseofulvin; 0.12 µg/mL for voriconazole and amorolfine; and 0.016 µg/mL for terbinafine (Fig. 3A through H). All isolates remained at or below the provisional ECVs for ciclopirox olamine and voriconazole, whereas isolates above the provisional ECV were identified for griseofulvin (20.5%), amorolfine (1.8%), and terbinafine (1.3%).

## DISCUSSION

This multicenter study provides species-stratified antifungal susceptibility data for clinical isolates of *T. mentagrophytes* and *T. interdigitale* collected in China and proposes provisional ECVs derived from CLSI M38-generated MIC distributions. A central finding is that these two closely related dermatophytes did not exhibit identical MIC distributions at the species level. Seven of eight *T. mentagrophytes* combinations and five of the eight *T. interdigitale* combinations were suitable for provisional ECV estimation. The remaining combinations, including fluconazole for both species and itraconazole and posaconazole for *T. interdigitale*, were not retained because of non-unimodal or unstable MIC distributions. These findings underscore the importance of species-level analysis within the *T. mentagrophytes*/*T. interdigitale* species complex and indicate that complex-level reporting alone may obscure meaningful distributional differences.

For *T. mentagrophytes*, the most notable distributional feature was observed for fluconazole, which showed a non-unimodal MIC pattern with a distinct high-MIC subpopulation. We therefore did not assign a provisional ECV or classify isolates as NWT for this agent, because fitting a single wild-type distribution to a heterogeneous MIC pattern could generate an unstable cutoff. Voriconazole yielded a lower provisional ECV than itraconazole and posaconazole, consistent with previous dermatophyte susceptibility studies showing lower voriconazole MICs relative to itraconazole and/or posaconazole in *T. rubrum* and *T. mentagrophytes* collections (20–22). Because ECVs are derived from modeled MIC distributions, this likely reflects drug- and species-specific distributional behavior rather than an unexpected biological anomaly. Overall, most non-fluconazole agents showed distributions compatible with ECV determination, supporting overall *in vitro* activity in the present *T. mentagrophytes* collection.

For *T. interdigital*e, fluconazole also showed a complex distribution that was not suitable for stable single-population modeling. Itraconazole and posaconazole showed partly center-associated low-MIC shoulders; therefore, robust species-level provisional ECVs were not established for these agents. In contrast, voriconazole retained a distribution suitable for modeling and yielded no NWT isolates, indicating that azole MIC behavior was not uniform within *T. interdigitale* (23). Taken together, the complex fluconazole distributions observed in both species should not be attributed directly to fluconazole-driven selection alone. Although fluconazole has been used clinically for dermatophytosis (24), contemporary reviews generally emphasize terbinafine and itraconazole as more active or preferred systemic agents, and several susceptibility studies have reported comparatively high or variable fluconazole MICs among dermatophytes (25). Although historical or selected fluconazole exposure cannot be excluded, these patterns should be interpreted cautiously and should not be taken as direct evidence of fluconazole-driven selection.

Among the non-azole agents, amorolfine and griseofulvin were particularly informative in *T. interdigitale*. For amorolfine, the provisional ECV was lower for *T. interdigitale* than for *T. mentagrophytes* (0.12 and 0.25 µg/mL, respectively), with corresponding non-wild-type (NWT) fractions of 1.8% and 0.7%. This is more likely to reflect subtle differences in MIC distribution and cutoff positioning than a generalized reduction in susceptibility. For griseofulvin, the provisional ECV of 0.25 µg/mL delineated the main MIC distribution, whereas isolates at 0.5–1 µg/mL represented a higher-MIC NWT subpopulation, accounting for 20.5% of tested *T. interdigitale* isolates. These isolates were detected across multiple participating centers, making a single-center testing artifact less likely. In the absence of clinical breakpoints, this should be interpreted as an NWT signal rather than a clinical resistance. Across both species, terbinafine, a key systemic agent for dermatophyte treatment, showed low NWT rates (1.3% in both species), and no NWT isolates were detected for ciclopirox olamine, supporting retained overall *in vitro* activity in the current collection (26).

Differences between our findings and previous studies likely reflect differences in species composition, genotype background, geographic origin, sampling context, and testing methodology. Shaw et al. (27) reported higher wild-type limits for terbinafine, fluconazole, and griseofulvin in an Indian multicenter study that was effectively dominated by *T. indotineae*-related isolates and was not analyzed at the species level. Ebert et al. (28) similarly described an Indian multicenter collection dominated by internal transcribed spacer (ITS) type VIII/*T. indotineae* with high frequencies of antifungal resistance. The Iranian study (29) further supports geographic variation in species-level susceptibility patterns, particularly where high-terbinafine-MIC type VIII isolates are present. Todd et al. (30) also reported broad fluconazole MIC distributions in a New York collection comprising *T. indotineae*, *T. mentagrophytes* genotype VII, and *T. mentagrophytes* genotype II*. In comparison, our study used species-resolved contemporary clinical isolates from China and excluded *T. indotineae*, because inclusion of this distinct emerging species, which often contains a higher proportion of antifungal non-wild-type isolates, could artificially broaden the MIC distributions of *T. mentagrophytes* or *T. interdigitale* and affect species-level ECV determination (3, 31). Compared with the recent Chinese CLSI-based study, which focused on antifungal resistance characterization using centralized testing (21), our study was designed specifically for multicenter CLSI-based ECV determination using independently generated MIC data, allowing assessment of interlaboratory consistency under a shared protocol. The Chinese CARDS study (13) also differed from our routine clinical collection because it used centralized EUCAST-based testing in a refractory dermatophytosis-enriched population and reported increased itraconazole MICs in *T. mentagrophytes* and *T. interdigitale* (20.8% and 15.2%, respectively). Together, these comparisons underscore the need for species-resolved, method-specific, and geographically representative MIC data sets when establishing dermatophyte ECVs, with explicit attention to interlaboratory variation in MIC testing and differences in interpretive criteria.

The results should be interpreted within the appropriate ECV framework. ECVs are not clinical breakpoints and should not be used to directly predict treatment outcomes. Instead, their value lies in identifying isolates outside the modeled wild-type population and in supporting longitudinal surveillance of susceptibility shifts, as has been demonstrated for other medically important fungi, including *Candida* (32), *Cryptococcus* (33), and *Aspergillus* spp. (34). In this context, the low NWT proportion observed for most assigned agent-species combinations (≤2.5%) supports overall stability of the MIC distributions, whereas griseofulvin against *T. interdigitale* (20.5%) and combinations unsuitable for ECV estimation may require closer monitoring.

This study has several strengths. It was based on clinical isolates from six tertiary hospitals, providing a broader representation of contemporary strains than a single-center data set. All isolates were identified to the species level, allowing *T. mentagrophytes* and *T. interdigitale* to be analyzed separately, and each participating center performed AFST independently under a shared quality control strategy. In addition, both study species typically produce abundant microconidia *in vitro*, which supported reproducible inoculum preparation under CLSI M38 conditions. Several limitations should also be noted. The isolates were obtained from tertiary hospitals in China, so the generalizability of these provisional ECVs requires evaluation in broader regional and international collections. We also did not further investigate the molecular basis of all isolates above the provisional ECVs, and the proposed ECVs remain provisional pending validation in larger multicenter data sets. Nonetheless, these limitations do not detract from the value of this study as a multicenter baseline for species-resolved dermatophyte susceptibility monitoring in China.

### Conclusion

In conclusion, this multicenter CLSI M38-based study proposes provisional ECVs for seven antifungal agents against *T. mentagrophytes* and for five against *T. interdigitale* in a contemporary Chinese clinical collection. Fluconazole for both species and itraconazole and posaconazole for *T. interdigitale* were not assigned provisional ECVs because of non-unimodal or unstable MIC distributions. The two species showed distinct MIC distribution profiles and differed in the frequency of isolates outside the modeled wild-type population, highlighting the limits of complex-level interpretation alone. These data provide a practical species-specific laboratory basis for recognizing potential non-wild-type isolates and a contemporary baseline for dermatophyte susceptibility monitoring in China. They may also support ongoing efforts to standardize ECV determination and build the evidence base for future interpretive criteria in dermatophytes.

## MATERIALS AND METHODS

### Isolate collection

This retrospective multicenter study included dermatophyte isolates recovered between 2019 and 2024 from six tertiary hospitals across geographically diverse regions of China, representing eastern, central, and western areas. All isolates were obtained from skin, nail, or hair specimens of patients with dermatophytosis and stored in local strain collections. Only one isolate per patient was included.

A total of 305 isolates were analyzed, including 153 *T. mentagrophytes* isolates and 152 *T. interdigitale* isolates. The numbers of isolates contributed by each center were as follows: Institute of Dermatology, Chinese Academy of Medical Sciences (43 *T. mentagrophytes* and 45 *T. interdigitale*), the First Affiliated Hospital of Guangxi Medical University (30 *T. mentagrophytes* and 30 *T. interdigitale*), Xijing Hospital (25 *T. mentagrophytes* and 25 *T. interdigitale*), Peking University First Hospital (20 *T. mentagrophytes* and 20 *T. interdigitale*), Wuhan No. 1 Hospital (20 *T. mentagrophytes* and 15 *T. interdigitale*), and the First Affiliated Hospital of Xinjiang Medical University (15 *T. mentagrophytes* and 17 *T. interdigitale*).

### Species identification

Species identification was performed in the participating laboratories after subculture on potato dextrose agar. Genomic DNA was extracted from each isolate, and the internal transcribed spacer (ITS) region was amplified using primers ITS1 and ITS4. Amplicons were sequenced bidirectionally, and forward and reverse reads were assembled into ITS consensus sequences. Initial sequence screening was performed by BLAST comparison against GenBank sequences to confirm dermatophyte identity and membership within the TMTISC. Because BLAST best-hit results alone may not reliably distinguish closely related taxa within the TMTISC, species-level assignment was further determined by sequence-based phylogenetic comparison with published TMTISC reference sequences (Fig. S1), including those described by Normand et al. (35) and Todd et al. (30). Only isolates confirmed as *T. mentagrophytes* or *T. interdigitale* were retained for the ECV analysis. *T. indotineae* isolates were excluded from the present ECV analysis because their distinct epidemiological and antifungal susceptibility profiles could distort species-specific MIC distributions and ECV calculation (31, 36).

### Antifungal susceptibility testing (AFST)

The *in vitro* activities of eight antifungal agents commonly used for dermatophytosis were evaluated: fluconazole (0.12 to 64 µg/mL), ciclopirox olamine (0.03 to 16 µg/mL), griseofulvin (0.016 to 8 µg/mL), itraconazole, voriconazole, posaconazole, and amorolfine (each at 0.004 to 2 µg/mL), and terbinafine (0.001 to 0.06 µg/mL).

AFST was performed according to the CLSI broth microdilution reference method for dermatophyte molds (CLSI M38) (14). Stock solutions of antifungal agents were prepared in dimethyl sulfoxide and further diluted in 1× RPMI 1640 medium. Conidial suspensions were adjusted to a final inoculum of 1 × 10^3^ to 3 × 10^3^ CFU/mL and inoculated into 96-well microdilution plates containing serial twofold drug dilutions. Plates were incubated at 35°C and read visually after 96 h. For all eight agents, the MIC endpoint was defined as the lowest drug concentration producing approximately 80% growth inhibition relative to the drug-free growth control. All MICs were read independently by two investigators. When discrepant results occurred, the plates were re-read jointly, and a consensus MIC was recorded.

Before testing clinical isolates, interlaboratory quality control was verified at all six centers. *Candida parapsilosis* ATCC 22019 and *T. interdigitale* MRL1957 (ATCC MYA-4439) were included as CLSI-recognized QC strains to verify the broth microdilution system, with 24-h MICs for ATCC 22019 and 96-h MICs for ATCC MYA-4439 interpreted according to the QC ranges listed in CLSI M38M51S, fourth edition (37). Two internal dermatophyte control strains, *T. interdigitale* CAMS-CCPM-D-05896 and CAMS-CCPM-D-05150, were also included for interlaboratory consistency monitoring and 96-h endpoint reading. The two CAMS-CCPM strains were well characterized by recent clinical isolates preserved at the CAMS Collection Center of Pathogenic Microorganisms; they were not CLSI-recognized QC or reference strains and were not used for ECV determination. Their internal MIC monitoring ranges are provided in Table S2. Each center performed quality control testing on two consecutive occasions before clinical isolate testing, and the control strains were included in each routine AFST run, with no more than 20 clinical isolates tested per run.

### Epidemiological cutoff values

Provisional ECVs were calculated for antifungal agent–species combinations with MIC distributions consistent with a unimodal wild-type population. In this study, the provisional ECV was defined as the upper limit of the modeled wild-type MIC distribution that encompassed 97.5% of isolates, in accordance with CLSI M57 principles.

MIC distributions were analyzed using an iterative statistical approach implemented in the ECOFFinder (version 2.1) program (15). As MICs were obtained using the CLSI M38 reference method, ECVs were estimated and interpreted according to the CLSI M57 guideline (18). Agent–species combinations with non-unimodal, heterogeneous, or otherwise unstable MIC distributions were considered unsuitable for reliable provisional ECV determination.

For each antifungal agent and species, the MIC range, modal MIC, MIC50, MIC90, geometric mean MIC, and the proportions of isolates at or below and above the provisional ECV were calculated.

### Statistical analysis

Clinical type was summarized as counts and percentages and compared between species using the *χ*^2^ test. Because some clinical variables were unavailable for a subset of isolates, analyses were performed on available cases only. All tests were two-sided, and a *P* value of <0.05 was considered statistically significant. Statistical analyses were performed using GraphPad Prism (version 10.1.2).

## Data Availability

The raw MIC distribution data are provided in the supplemental material. The ITS consensus sequences generated in this study have been deposited in GenBank under accession numbers PZ710626 to PZ710930.

## SUPPLEMENTAL MATERIAL

Fig. S1 jcm.00567-26-s0001.pdf ITS-based maximum-likelihood phylogenetic tree of study isolates and 28 published TMTISC reference sequences. Published TMTISC reference sequences are highlighted in blue and labeled with accession numbers and published genotype designations.

Supplemental legends jcm.00567-26-s0002.docx Legends for Fig. S1 and Tables S1 and S2.

Table S1 jcm.00567-26-s0003.docx MIC distribution counts for eight antifungal agents against *T. mentagrophytes* and *T. interdigitale*.

Table S2 jcm.00567-26-s0004.docx Internal MIC monitoring ranges for two dermatophyte control strains used in interlaboratory consistency assessment.

ASM does not own the copyrights to Supplemental Material that may be linked to, or accessed through, an article. The authors have granted ASM a non-exclusive, world-wide license to publish the Supplemental Material files. Please contact the corresponding author directly for reuse.
