# Supplementary material for: In vitro susceptibility and provisional epidemiological cutoff values for eight antifungal agents against Trichophyton mentagrophytes and Trichophyton interdigitale: a multicenter study from China
Source: J Clin Microbiol. 2026 Aug 21;64(9):e00567-26. doi: 10.1128/jcm.00567-26 (PMC13556232; doi:10.1128/jcm.00567-26)
Supplement: Supplementary file 1 [file jcm.00567-26-s0001.pdf]

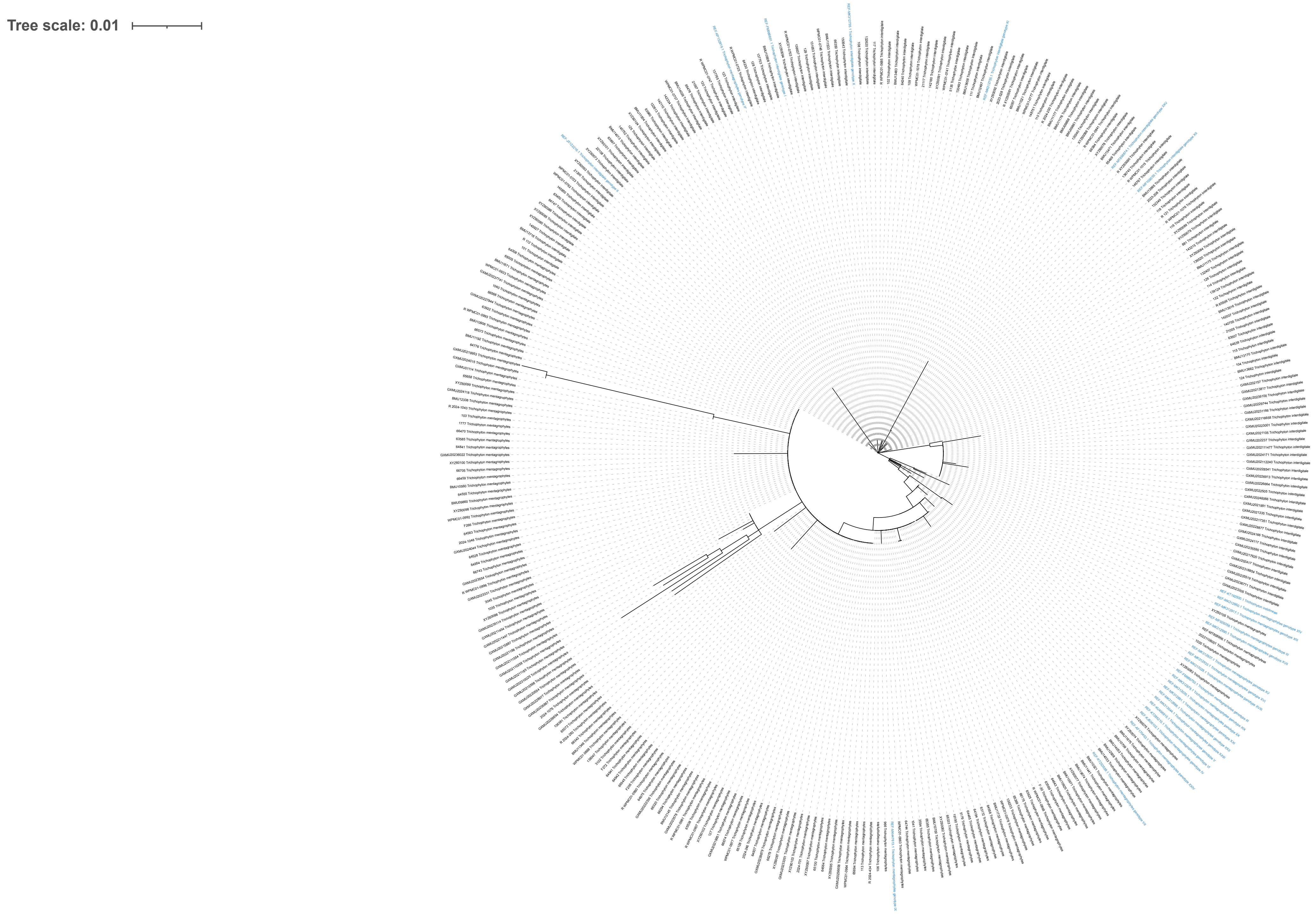
Tree scale: 0.01
