# Supplementary material for: In vitro susceptibility and provisional epidemiological cutoff values for eight antifungal agents against Trichophyton mentagrophytes and Trichophyton interdigitale: a multicenter study from China
Source: J Clin Microbiol. 2026 Aug 21;64(9):e00567-26. doi: 10.1128/jcm.00567-26 (PMC13556232; doi:10.1128/jcm.00567-26)
Supplement: Supplementary file 2 [file jcm.00567-26-s0002.docx]

**Supplementary**

Supplementary Table S1. MIC distribution counts for eight antifungal agents against *T. mentagrophytes* and *T. interdigitale*.

Supplementary Table S2. Internal MIC monitoring ranges for two dermatophyte control strains used in interlaboratory consistency assessment.

Supplementary Fig. S1. ITS-based maximum-likelihood phylogenetic tree of study isolates and 28 published TMTISC reference sequences. Published TMTISC reference sequences are highlighted in blue and labeled with accession numbers and published genotype designations.
