# Supplementary material for: In vitro susceptibility and provisional epidemiological cutoff values for eight antifungal agents against Trichophyton mentagrophytes and Trichophyton interdigitale: a multicenter study from China
Source: J Clin Microbiol. 2026 Aug 21;64(9):e00567-26. doi: 10.1128/jcm.00567-26 (PMC13556232; doi:10.1128/jcm.00567-26)
Supplement: Supplementary file 3 [file jcm.00567-26-s0003.docx]

| **Species** | **Antifungal agent (n)** | **Number of isolates per minimum inhibitory concentrations (μg/mL)** | | | | | | | | | | | | | | | | |
| --- | --- | --- | --- | --- | --- | --- | --- | --- | --- | --- | --- | --- | --- | --- | --- | --- | --- | --- |
|  |  | **0.001** | **0.002** | **0.004** | **0.008** | **0.016** | **0.03** | **0.06** | **0.12** | **0.25** | **0.5** | **1** | **2** | **4** | **8** | **16** | **32** | **64** |
| *T. mentagrophytes* | Fluconazole (103) |  |  |  |  |  |  |  | 1 | 11 | 33 | 22 | 4 | 5 | 11 | 13 | 3 |  |
|  | Ciclopirox Olamine (128) |  |  |  |  |  |  | 3 | 28 | 62 | 35 |  |  |  |  |  |  |  |
|  | Griseofulvin (153) |  |  |  |  |  |  | 2 | 3 | 40 | 92 | 16 |  |  |  |  |  |  |
|  | Itraconazole (118) |  |  |  | 1 |  | 1 | 2 | 44 | 59 | 11 |  |  |  |  |  |  |  |
|  | Voriconazole (153) |  |  | 2 | 32 | 57 | 48 | 11 | 3 |  |  |  |  |  |  |  |  |  |
|  | Posaconazole (108) |  |  |  | 1 | 3 | 6 | 40 | 38 | 18 | 2 |  |  |  |  |  |  |  |
|  | Amorolfine (133) |  |  | 2 | 3 | 14 | 35 | 39 | 35 | 4 | 1 |  |  |  |  |  |  |  |
|  | Terbinafine (153) | 4 | 15 | 68 | 43 | 21 | 1 | 1 |  |  |  |  |  |  |  |  |  |  |
| *T. interdigitale* | Fluconazole（152） |  |  |  |  |  |  |  | 13 | 6 | 12 | 10 | 15 | 11 | 52 | 31 | 2 |  |
|  | Ciclopirox Olamine (127) |  |  |  |  |  | 6 | 8 | 43 | 66 | 4 |  |  |  |  |  |  |  |
|  | Griseofulvin (122) |  |  |  |  | 1 | 7 | 25 | 52 | 12 | 22 | 3 |  |  |  |  |  |  |
|  | Itraconazole (120) |  |  | 4 | 2 | 5 | 3 | 6 | 30 | 65 | 5 |  |  |  |  |  |  |  |
|  | Voriconazole (135) |  |  | 3 | 8 | 25 | 50 | 41 | 8 |  |  |  |  |  |  |  |  |  |
|  | Posaconazole (120) |  |  | 5 | 2 | 4 | 7 | 26 | 48 | 24 | 4 |  |  |  |  |  |  |  |
|  | Amorolfine (112) |  |  | 8 | 4 | 15 | 38 | 29 | 16 | 1 | 1 |  |  |  |  |  |  |  |
|  | Terbinafine (152) | 28 | 30 | 57 | 30 | 5 | 2 |  |  |  |  |  |  |  |  |  |  |  |

**Supplementary Table S1. MIC distribution counts for eight antifungal agents against *T. mentagrophytes* and *T. interdigitale*.**
