# Supplementary material for: In vitro susceptibility and provisional epidemiological cutoff values for eight antifungal agents against Trichophyton mentagrophytes and Trichophyton interdigitale: a multicenter study from China
Source: J Clin Microbiol. 2026 Aug 21;64(9):e00567-26. doi: 10.1128/jcm.00567-26 (PMC13556232; doi:10.1128/jcm.00567-26)
Supplement: Supplementary file 4 [file jcm.00567-26-s0004.docx]

| **Antifungal agent** | **CAMS-CCPM-D-05896 MIC range (μg/mL)** | **CAMS-CCPM-D-05150 MIC range (μg/mL)** |
| --- | --- | --- |
| Fluconazole | 4–32 | 2–8 |
| Ciclopirox olamine | 0.12–0.25 | 0.12–0.5 |
| Griseofulvin | 0.12–1 | 0.06–0.25 |
| Itraconazole | 0.06–0.5 | 0.016–0.12 |
| Voriconazole | 0.03–0.12 | 0.016–0.06 |
| Posaconazole | 0.03–0.25 | 0.004–0.03 |
| Amorolfine | 0.016–0.12 | 0.008–0.06 |
| Terbinafine | 0.002–0.008 | 0.001–0.002 |

**Supplementary Table S2. Internal MIC monitoring ranges for dermatophyte control strains used for interlaboratory consistency assessment.**

CAMS-CCPM-D-05896 and CAMS-CCPM-D-05150 were internal dermatophyte control strains and were not CLSI-recognized QC or reference strains. These ranges were used for interlaboratory consistency monitoring and 96-h dermatophyte endpoint reading, and not for ECV determination.
